# Investigation of thermal energy transport interface of hybrid graphene-carbon nanotube/polyethylene nanocomposites

**DOI:** 10.1038/s41598-017-14710-4

**Published:** 2017-10-31

**Authors:** Feng Liu, Xuyang Liu, Ning Hu, Huiming Ning, Satoshi Atobe, Cheng Yan, Fuhao Mo, Shaoyun Fu, Jianyu Zhang, Yu Wang, Xiaojing Mu

**Affiliations:** 1 College of Aerospace Engineering, Chongqing University, Chongqing, 400044 China; 2 The State Key Laboratory of Mechanical Transmissions, Chongqing University, Chongqing, 400044 China; 3 Department of Aerospace Engineering, Tohoku University, 6-6-01 Aramaki-aza-Aoba, Aoba-ku, Sendai, 980-8579 Japan; 4 School of Chemistry, Physics and Mechanical Engineering, Queensland University of Technology (QUT), Brisbane, QLD 4001 Australia; 5 College of Mechanical and Vehicle Engineering, Hunan University, Changsha, 410082 China; 6 School of Chemistry and Chemical Engineering, Chongqing University, Chongqing, 401331 China; 7 Key Disciplines Lab of Novel Micro-nano Devices and System Technology, International R&D center of Micro-nano Systems and New Materials Technology, Chongqing University, Chongqing, 400044 China

## Abstract

It is well known the thermal properties of three-dimensional (3-D) hybrid graphene (GR)-carbon nanotube (CNT) structures are not superior to that of the individual GR and CNT, however, the 3-D hybrid GR-CNT structures can effectively improve the thermal properties of polymer matrix. Therefore, understanding the thermal energy transport in the interface between polymer matrix and 3-D hybrid GR-CNT structure is essential. Here, the enhancement mechanism of interfacial thermal transport of hybrid GR-CNT structure was explored by applying non-equilibrium molecular dynamics (NEMD) simulations. Three different types of hybrid GR-CNT structures were built. The influences of CNT radius and CNT type for the hybrid GR-CNT on the interfacial thermal properties were also analyzed. Computational results show that among the three different types of hybrid GR-CNT structures, the Model-I, i.e., the covalent bond hybrid GR-CNT structures are of the best interfacial thermal properties. Meanwhile, the CNT radius of hybrid GR-CNT structure has a great influence on the interfacial thermal properties.

## Introduction

The interfacial thermal resistance is critical to nanocomposites and can significantly limit the thermal conductivity of nanocomposites^1^. Therefore, the interfacial thermal transport between nanofillers and matrices has been widely studied^2–6^.

Carbon nanotube (CNT) and graphene (GR) both have extremely high thermal conductivity, e.g., ~3500 W/mK for single-walled CNTs (SWCNTs)^7^ and ~3000 W/mK for multi-walled CNTs (MWCNTs)^8^, while GR is in the range of ~3080–5300  W/mK^9,10^. Investigations indicate that introducing CNTs and GR into matrices can effectively enhance the thermal conductivity of the matrices^11–13^. However, the increase of thermal conductivity of polymer is still modest, due to the effect of interfacial thermal resistance^5,6,13,14^. A possible way to reduce the interfacial resistance between nanofillers and matrix is to functionalize the nanofillers^4,15–18^.

GR and CNT have outstanding thermal properties, however, only limited in the in-plane direction and axis direction of GR and CNT, respectively^19–24^. In order to take full advantage of the thermal properties of the two carbon materials, a 3-D hybrid GR-CNT structure can be fabricated through combining the CNT and the GR. Investigations show that the 3-D hybrid GR-CNT structure has the promising high thermal properties of both the individual GR and CNT^21,25–27^. Although the thermal conductivity of 3-D hybrid GR-CNT structure is lower than that of its carbon based constituent materials (i.e., CNT and GR)^26,28^, especially in in-plane direction (i.e., along the GR plane)^21^, this structure has more contact areas with matrix than that of GR and CNT with matrix. Therefore, it can effectively transport thermal energy from matrix to the 3-D hybrid GR-CNT structure.

Owing to their outstanding thermal properties, the hybrid GR-CNT materials are widely used to improve the thermal conductivity of polymer matrix. From experimental results, Yang *et al*.^29^ found that the thermal conductivity of polymer matrix can be effectively enhanced after the hybrid GR-MWCNT structure was introduced. The reason is that this 3-D hybrid structure has more efficient percolating nanoparticle networks with significant reduced thermal interface resistances^30^. Su *et al*.^31^ found that non-covalent bond hybrid structure can improve the thermal conductivity of epoxy composites to about 2 times of neat epoxy. Although the hybrid GR-CNT structure can effectively improve the thermal conductivity of polymer matrix, the mechanism of interfacial thermal transport between this structure and polymer matrix has not been explored and well understood.

In this work, the thermal energy transport across the interface of hybrid GR-CNT structure and polyethylene (PE) matrix was systematically investigated using non-equilibrium molecular dynamics (NEMD)^32–34^ simulations. The influences of three different hybrid GR-CNT structures with different CNT radii and CNT types on the interfacial thermal transport between the hybrid GR-CNT structures and the PE matrices were investigated. The computational results indicated that the covalent bond hybrid GR-CNT structure has better interfacial thermal properties than that of the other two non-covalent bond hybrid GR-CNT structures.

## Results and Discussion

### Effect of model size

In order to analyze the effect of simulation model size on the results, the length of the simulation cell in the *x*-axis (i.e., heat flux direction) from 57 Å to 220 Å was used to investigate the thermal conductivity of neat PE matrix. And the thermal conductivity can be obtained using the Fourier’s law as

1 $${J}_{Q}=-\kappa \frac{{\rm{\Delta }}T}{{\rm{\Delta }}x}$$

where ${J}_{Q}$ is the heat flux through the system, $\kappa$ is the thermal conductivity, and ${\rm{\Delta }}T/{\rm{\Delta }}x$ is the temperature gradient along the *x*-axis.

The heat flux ${J}_{Q}$ can be obtained as^2^

2 $${J}_{Q}=\frac{Q}{2At}$$

where *Q* is the amount of energy imposed on the system in a given times, *A* is the cross section area of system and *t* is the total simulation time, and the factor 2 arises because of the periodic boundary condition of the system in the heat flux direction.

**Figure 1:**
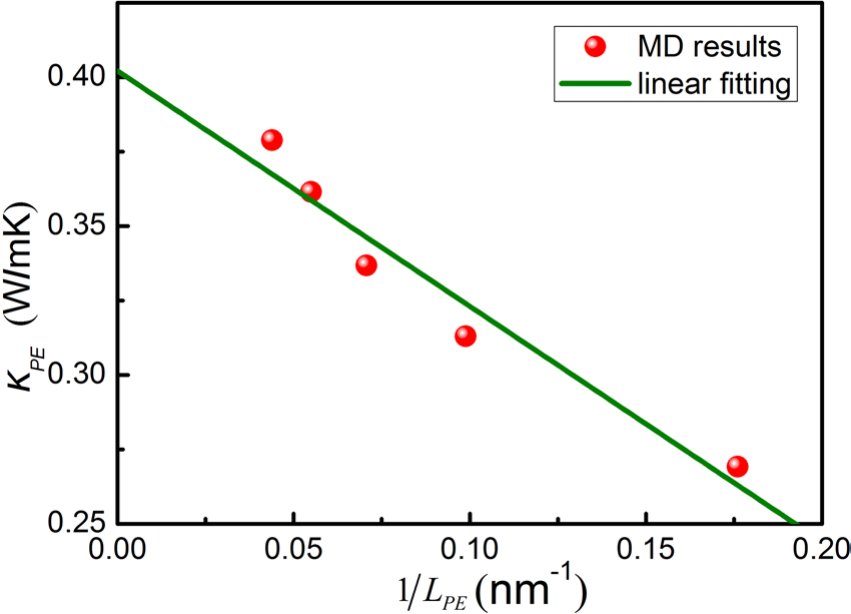
Thermal conductivity of PE models as a function of the inverse of model length.

Figure 1 shows the computational results as a function of the inverse of model length. Generally, the theoretical thermal conductivity of PE matrix can be calculated by using Eq. (3)^26,35^, and the thermal conductivity for an infinitely large sample can be obtained by simple extrapolation, i.e., ${L}_{PE}\to \infty (1/{L}_{PE}\to 0)$. Therefore, through linear fitting of the computational results we obtained the thermal conductivity of neat PE matrix as 0.402 W/mk, which is consistent with theoretical result of ~0.41 W/mk^17,36–38^.

3 $$\frac{1}{{\kappa }_{PE}}=\frac{1}{{l}_{PE}}+\frac{1}{{L}_{PE}}$$

where ${\kappa }_{PE}$ is the thermal conductivity of PE matrix, ${l}_{PE}$ is the phonon mean free path (MFP) for an infinitely long system, and ${L}_{PE}$ is the length of PE matrix.

Based on the above computational results, it indicated that the simulation results were reasonable and close to the theoretical result (~0.41 W/mk) when the length of simulation cell is longer than 220 Å. To obtain more accurate results, the length of ~360 Å for the simulation cell was applied for all the simulation systems.

### Interfacial thermal conduction

Interfacial resistance (or interfacial conductance) is critical in the thermal transport of nanocomposites^39,40^, it can affect the overall thermal properties of nanocomposites. Using Muller-Plathe method we can calculate the interfacial thermal properties between nanofillers and polymer matrix by introducing both heat source and heat sink in the simulation models. And the interfacial thermal conductance ${G}_{k}$ is given by

4 $${G}_{k}=-\frac{{J}_{Q}}{{\rm{\Delta }}T}$$

where ${\rm{\Delta }}T$ is a discontinuous temperature drop at the interface.

Based on the Eq. (4), the interfacial thermal conductance of GR/PE nanocomposites can be calculated and that is 51.03 MW/m^2^K, which is in a reasonable range for various GR-polymer nanocomposites^4,17^. The simulation result also indicates that our computational systems are reasonable.

A 3-D hybrid GR-CNT structure can be fabricated using chemical vapor deposition (CVD)^41–43^ or mixing GR and CNTs^44,45^. Therefore, the former method is usually used to synthesize covalent bond hybrid GR-CNT structures and the latter method is used to create non-covalent bond hybrid GR-CNT structures. Meanwhile, Liu *et al*.^46^ found that the interfacial mechanical properties of non-covalent bond 3-D hybrid GR-CNT structures were different from the covalent bond 3-D hybrid GR-CNT structures. Therefore, it is possible that the interfacial thermal properties of these two hybrid GR-CNT structures are different, and needed to be explored.

**Figure 2:**
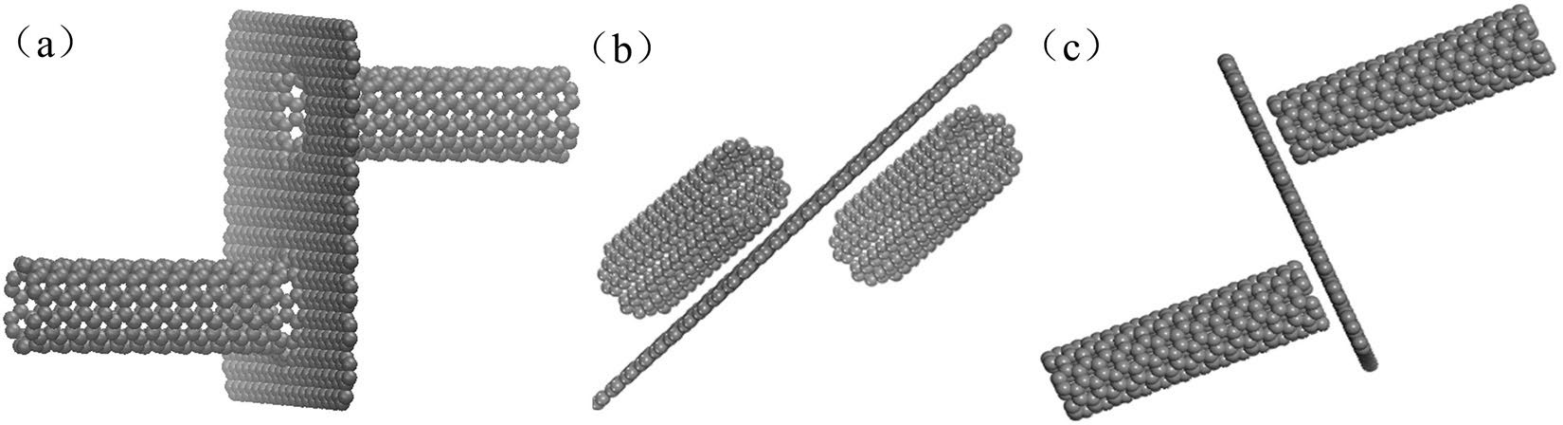
Three types of hybrid GR-CNT structures. (**a**) Model-I covalent bond connection. (**b**) Model-II non-covalent bond connection. (**c**) Model-III non-covalent bond connection.

Based on the above discussion, in this work, we constructed three types of hybrid GR-CNT structures, as shown in Fig. 2. In Model-I the CNTs are connected with GR using C-C covalent bonds (Fig. 2(a)). For the non-covalent bond hybrid GR-CNT structures, CNTs are connected with GR by the van der Waals (vdW) and Coulomb interactions. In all hybrid GR-CNT structures, the CNTs and the GRs are not passivated with hydrogen atoms. Among these non-covalent bond hybrid structures, the CNTs are parallel to the GR in Model-II (Fig. 2(b)), and in Model-III the CNTs are perpendicular to the GR (Fig. 2(c)). More detailed hybrid GR-CNT structures can be seen in Figure S1 (Supplementary information).

**Figure 3:**
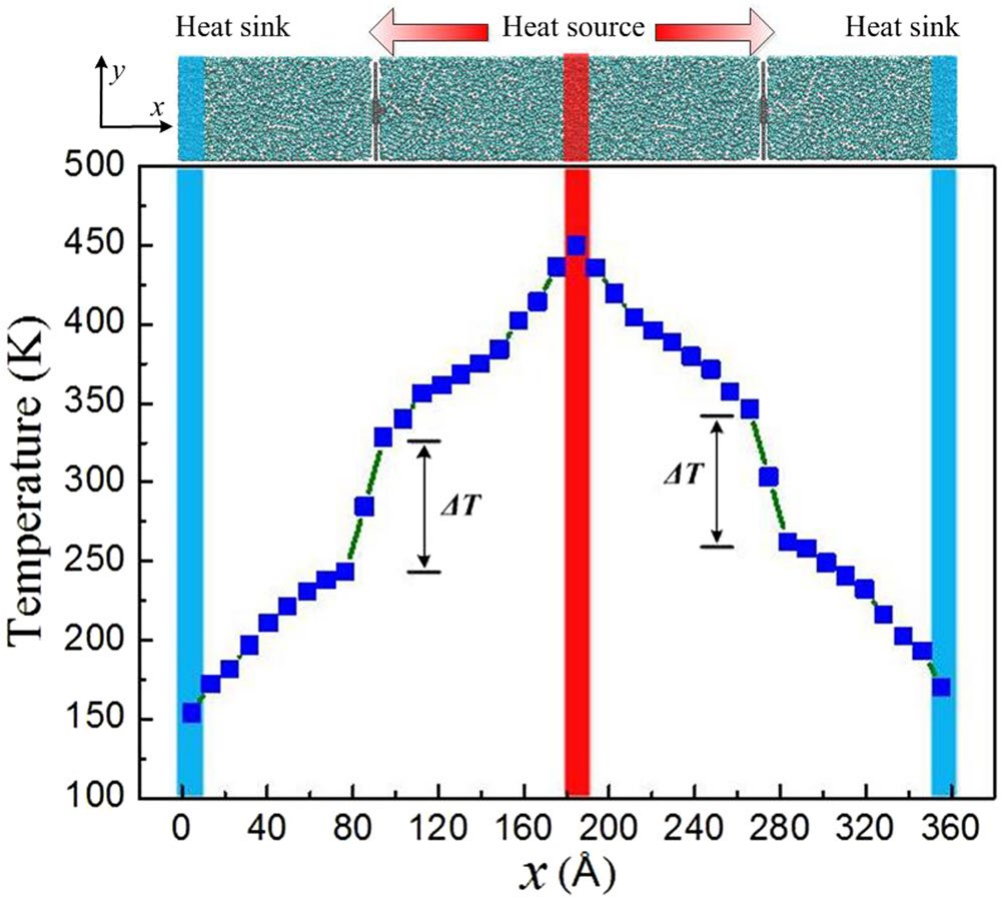
Model of covalent bond hybrid GR-CNT/PE nanocomposite (top panel) and the corresponding steady-state temperature profile (bottom panel). The scenograph of GR-CNT/PE nanocomposite can be seen in Figure S2 (Supplementary information).

In order to conveniently study the interfacial thermal properties of hybrid GR-CNT/PE nanocomposites, we regard the CNTs as functional groups and use the temperature drops between the GR and PE matrix to calculate the interfacial thermal properties between the hybrid GR-CNT structure and PE matrix. Using NEMD simulation and a long time NVE ensemble relaxation, we can obtain a steady-state temperature profile of covalent bond hybrid GR-CNT/PE nanocomposites, as shown in the bottom panel of Fig. 3. It is shown that an obvious temperature discontinuity (${\rm{\Delta }}T$) arises at the GR/PE interface, suggesting a significant interfacial thermal resistance across the interface of hybrid GR-CNT structure and PE matrix.

**Figure 4:**
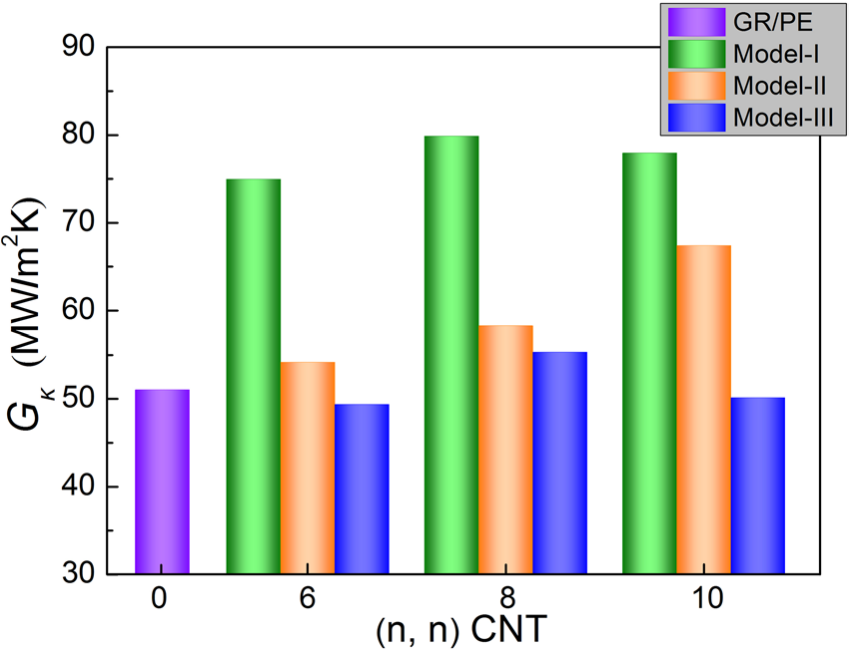
Interfacial thermal conductance *G*_*κ*_ of hybrid GR-SWCNT/PE nanocomposites as a function of CNT radius for three types hybrid models.

Figure 4 gives the variation of interfacial thermal conductance between PE matrix and three different types of hybrid GR-SWCNT structures as a function of CNT radius (three different radii were used, i.e., 4.07, 5.43 and 6.78 Å for (6, 6), (8, 8) and (10, 10) armchair SWCNTs, respectively). It is obvious that Model-I possesses the best interfacial thermal conductance compared with the other two models. It indicates that covalent bond hybrid GR-CNT structures have better thermal properties than that of non-covalent bond hybrid GR-CNT structures, which is consistent with the conclusion in ref.^25^, which is only for hybrid nanofillers. It was found that CNT connected with GR using covalent bonds has a lower thermal resistance in the junctions than that of CNT connected with GR using non-covalent bonds^25^, i.e., van der Waals. For Model-I and Model-III, the ${G}_{k}$ increases with CNT radius and then decreases. One possible reason is that the phonon scattering in the hybrid GR-CNT junctions increases with the increase of CNT radius, due to the lattice mismatching in the hybrid GR-CNT junctions. Compared with GR, the ${G}_{k}$ of Model-I with (8, 8) CNT increases 56.5%. For Model-II, the ${G}_{k}$ monotonously increases with CNT radius. It is probably attributed to the fact that CNTs are parallel to the GR, and the increase of CNT radius leads to more carbon atoms of CNT interacting with the carbon atoms of GR, which is beneficial to the kinetic energy transport. We can also observe that a small or large CNT radius may cause the ${G}_{k}$ of Model-III weaker than that of GR/PE nanocomposites.

### Effect of CNT type

**Figure 5:**
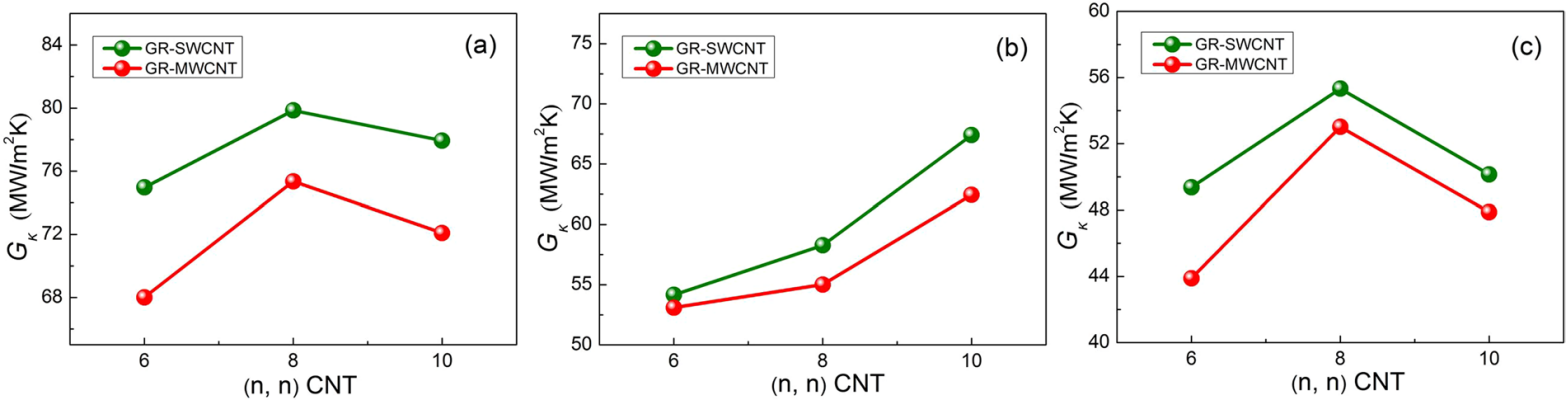
Variation of *Gκ* of hybrid GR-SWCNT/PE and GR-MWCNT/PE nanocomposites with CNT radius. (**a**) Model-I covalent bond hybrid GR-CNT. (**b**) Model-II non-covalent bond hybrid GR-CNT. (**c**) Model-III non-covalent bond hybrid GR-CNT.

Some previous investigations have found that SWCNTs are different with MWCNTs in thermal transport behaviors. The thermal conductivities of SWCNTs and MWCNTs are 2400 MW/mK and 1400 MW/mK^47^, respectively, or ~3500 W/m Kfor SWCNTs^7^ and ~3000 for MWCNTs^8^. During the fabrication process, CNTs are usually synthesized as multi-walled structures^41,48–50^. Therefore, it is essential to investigate the interfacial thermal properties of PE matrix with 3-D hybrid GR-MWCNT structures, and the computational results are shown in Fig. 5. Note that three independent MWCNTs used in simulations are double-walled CNTs. Their outermost walls are (6, 6), (8, 8) and (10, 10) armchair SWCNTs, the corresponding inner walls are (3, 3), (5, 5) and (7, 7) armchair SWCNTs, respectively. (*n*, *n*) in the *x*-axis of Fig. 5 represents their outermost walls. It shows that the variation trends of ${G}_{k}$ for hybrid GR-MWCNT/PE nanocomposites are similar to the hybrid GR-SWCNT/PE nanocomposites. However, the ${G}_{k}$ of hybrid GR-MWCNT/PE nanocomposites are smaller than that of the hybrid GR-SWCNT/PE nanocomposites. The reason is that the thermal properties of SWCNTs are much better than those of MWCNTs^8,51^, as the interlayer interactions can reduce the thermal conductivity of MWCNTs^51^.

**Figure 6:**
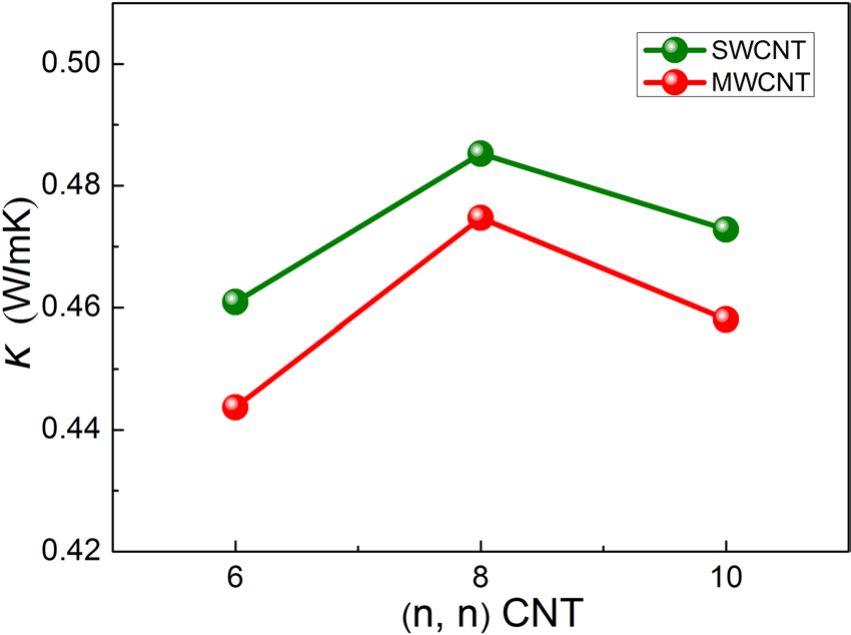
Thermal conductivity of CNT/PE nanocomposites with different CNT radii.

In this work, we also calculated the thermal conductivity of CNT/PE nanocomposites along the CNT axis, and the computational results can be seen in Fig. 6. Except for the CNT length, the wall number of CNT and the way of CNT introduced into the PE matrix are similar to the hybrid GR-CNT structures. It reveals that the thermal conductivity of SWCNT/PE nanocomposites is better than that of MWCNT/PE nanocomposites, this result is consistent with other research work^52^ as the thermal conductivity of SWCNTs is much better than that of MWCNTs^8,51^. Figure 6 also suggests that the thermal conductivity of CNT/PE nanocomposites increases with CNT radius and then decreases after the radius being larger than 5.43 Å, i.e., for (8, 8) CNT, and this variation trend is similar to the Fig. 5(a) and (c). This result also can explain the variation trend of ${G}_{k}$ of Model-I and Model-III with different CNT radii in Fig. 4.

### Vibration power spectrum analysis

Generally, the kinetic energy transport across the interfaces between the atoms of nanofillers and those of matrix across the interfaces governs the thermal properties of nanocomposites. In order to understand the enhancement mechanism of ${G}_{k}$ for the covalent bond hybrid GR-CNT, i.e., GR connected with CNT using C-C covalent bonds, the vibration power spectra (VPS) of GR and hybrid GR-CNT were used to study the interfacial thermal energy transport. Using the discrete Fourier transform (FT) of the velocity autocorrelation functions (VAF), we can obtain the VPS at frequency $\omega$ as

5 $$D(\omega )={\int }_{0}^{\tau }{\rm{\Gamma }}(t)\cos (\omega t)dt$$

And the velocity autocorrelation functions ${\rm{\Gamma }}(t)$ of atoms can be defined as

6 $${\rm{\Gamma }}(t)=\langle v(t)\cdot v(0)\rangle $$

where $v(t)$ is the velocity of atoms at time *t*, $v(0)$ is the velocity of atoms at time 0.

**Figure 7:**
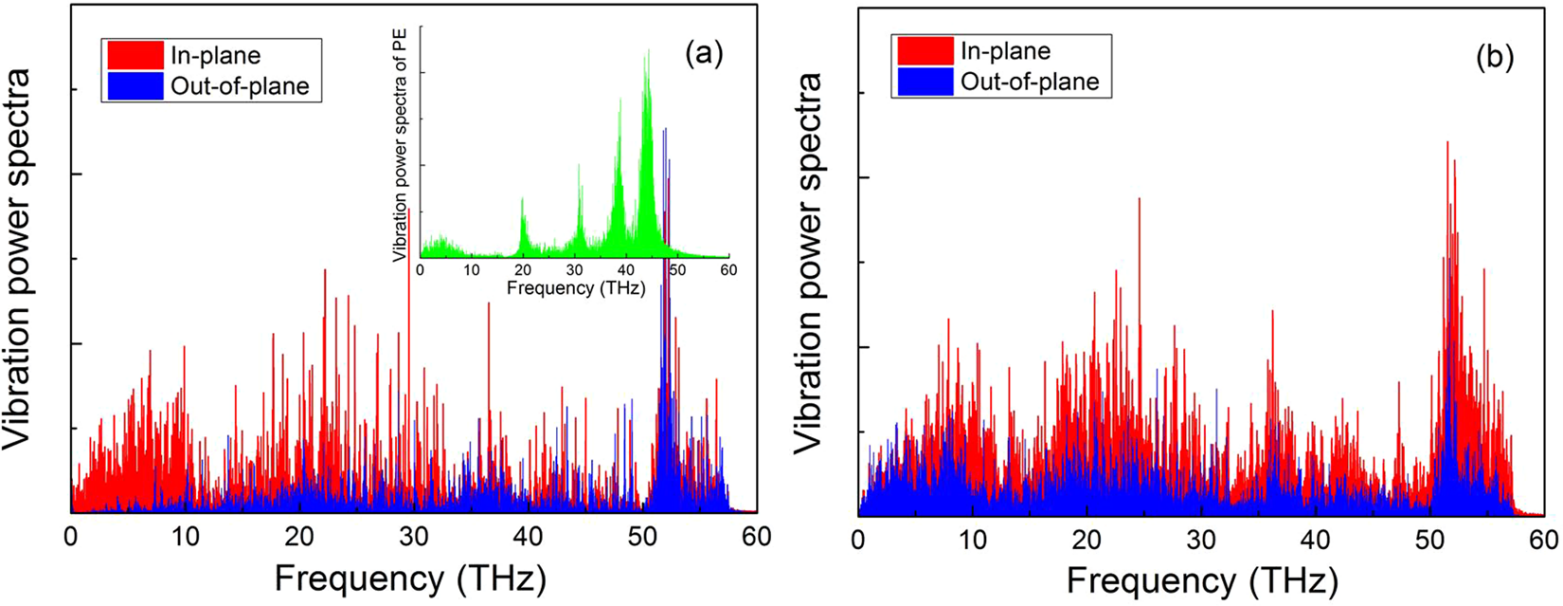
In-plane and out-of-plane VPS of nanofillers. (**a**) GR (inset is the VPS of PE matrix). (**b**) Covalent bond hybrid GR-SWCNT structure.

GR and hybrid GR-CNT are highly anisotropic materials, therefore their VPS can be decomposed into in-plane and out-of-plane spectra, as shown in Fig. 7. It is seen from Fig. 7(a) that the in-plane and out-of-plane VPS of GR are mainly distributed in 0–57 THz and 10–57 THz, respectively. For the hybrid GR-CNT structure, the distribution ranges of in-plane VPS are similar with GR and in 0–57 THz, however, the distribution range of out-of-plane VPS extends to 0–57 THz, as shown in Fig. 7(b). Since the VPS of PE matrix are mainly distributed in 0–10 THz and 20–50 THz, as shown in the inset of Fig. 7(a), the redistribution of out-of-plane VPS of hybrid GR-CNT increases the overlap range with the VPS of PE matrix (0–10 THz), leading to the increase of ${G}_{k}$ of hybrid GR-CNT/PE nanocomposites.

## Conclusions

In this work, the interfacial thermal properties of three different types of hybrid GR-CNT structures with PE matrix were analyzed using NEMD simulations, and the effects of CNT radius and CNT type were also investigated. The ${G}_{k}$ of Model-I and Model-III increases with CNT radius and then decreases, however, the ${G}_{k}$ of Model-II monotonously increases with CNT radius. The covalent bond hybrid GR-SWCNT structure has the best interfacial thermal property. Compared with GR, it also performs much better and the improvement ratio of ${G}_{k}$ is up to 56.5%. The out-of-plane VPS of the covalent bond hybrid GR-SWCNT structure is redistributed and extended to 0–57 THz, leading to its more VPS overlap with that of PE matrix. Analysis also shows that the hybrid GR-SWCNT structures have better interfacial thermal properties than those of hybrid GR-MWCNT structures.

## Methods

### Simulation model

In this work, polyethylene (PE) was used as the matrix and the PE molecular consists of 40 (-CH_2_-CH_2_-) repeated units, which is long enough to obtain stable results^53–55^. The cross section area of initial simulation cell was 47 Å × 45 Å for the simulation system, and the width (*W*) and length (*L*) of GR were 41.2 Å and 44.3 Å, respectively. For the hybrid structure, armchair CNTs of length of 32 Å were used, initially. Then CNT length was extended to 61.5 Å for the calculation of the thermal conductivity of CNT/PE nanocomposites along the CNT axis direction. In all the simulation systems periodic boundary conditions (PBCs) were applied in all three coordinate-axis directions. All of atomistic models were constructed using Materials Studio. For conveniently applying the NEMD to calculate the thermal properties of simulation systems, all simulation models along the *x*-axis were equally divided into 41 slices.

### Force fields

In this paper, the molecular dynamics simulations were carried out using LAMMPS molecular dynamics package. The *ab initio* polymer consistent force field (PCFF) was used to describe the interatomic interactions. The PCFF force field can be used to perform the interfacial thermal motivation between the carbon structures and the polymer matrices and it has been successfully applied to explore the thermal properties of GR systems and CNT systems^15,17,56^. The cut-off distance for both the van der Waals (vdW) and Coulomb interactions was 1.0 nm in all simulation systems.

### Simulation process

All the simulation models were first relaxed in isothermal-isobaric (NPT) ensemble at temperature of 300 K and pressure of 1 atm for 1.5 ns with the time step of 1 fs. Then the NEMD simulations with Muller-Plathe algorithm^57^ were performed, by introducing both heat source and heat sink in the middle and two ends of simulation models, respectively, as shown in the top panel of Fig. 3. A temperature gradient in the simulation model along the heat flux direction will be created through swapping the kinetic energy of coldest atoms in the heat source and the hottest atoms in the heat sink. After about 2 ns computation in NVE stable results can be obtained. Additionally, further simulation of 2000 ps was run with the same ensemble and 5 configurations were obtained every 400 ps to average the computational results.

## Electronic supplementary material

Supplementary information
