# Supplementary material for: Investigation of thermal energy transport interface of hybrid graphene-carbon nanotube/polyethylene nanocomposites
Source: Sci Rep. 2017 Oct 31;7:14700. doi: 10.1038/s41598-017-14710-4 (PMC5666017; doi:10.1038/s41598-017-14710-4)
Supplement: Supplementary file 1 — Supplementary information [file 41598_2017_14710_MOESM1_ESM.doc]

**Investigation of thermal energy transport interface of hybrid**

**graphene-carbon nanotube/polyethylene nanocomposites**

Feng Liu^1^, Xuyang Liu^1^, Ning Hu^1^,^2^,*, Huiming Ning^1^,*, Satoshi Atobe^3^, Cheng Yan^4^, Fuhao Mo^5^, Shaoyun Fu^1^, Jianyu Zhang^1^, Yu Wang^6^ and Xiaojing Mu^2^

^1^College of Aerospace Engineering, Chongqing University, Chongqing, 400044, China

^2^Key Disciplines Lab of Novel Micro-nano Devices and System Technology, International R&D center of Micro-nano Systems and New Materials Technology, Chongqing University, Chongqing, 400044, China

^3^Department of Aerospace Engineering, Tohoku University, 6-6-01 Aramaki-aza-Aoba, Aoba-ku, Sendai 980-8579, Japan

^4^School of Chemistry, Physics and Mechanical Engineering, Queensland University of Technology (QUT), Brisbane, QLD 4001, Australia

^5^College of Mechanical and Vehicle Engineering, Hunan University, Changsha, 410082, China

^6^School of Chemistry and Chemical Engineering, Chongqing University, Chongqing, 401331, China


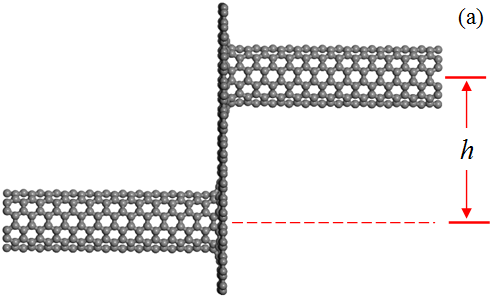

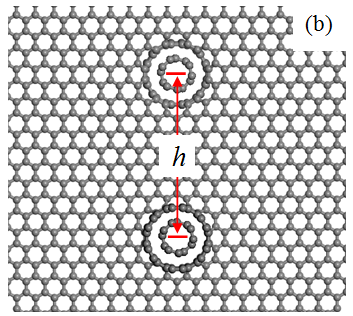


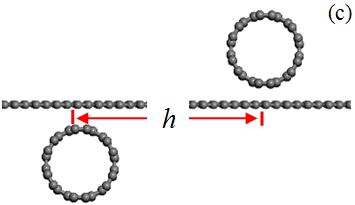

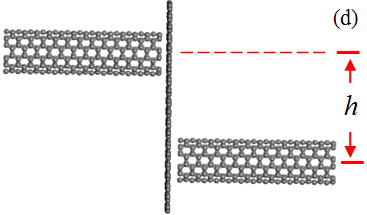


Figure S1. The hybrid GR-CNT structures (a) GR-SWCNT of Model-I, (b) GR-MWCNT of Model-I. (c) GR-SWCNT of Model-II, (d) GR-SWCNT of Model-III. The *h* is the distance of CNTs’ center, and it is a constant in all hybrid GR-CNT structures. In Fig. S1(b) the outermost walls of MWCNTs are covalent bonded with GR.


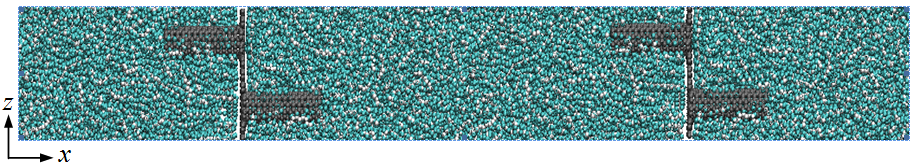


Figure S2. The scenograph of covalent bond hybrid GR-CNT/PE nanocomposite.

Table 1 The interfacial thermal conductance of GR/PE and Model-I systems.

|  |  | *G_k_* (MW/m^2^K) |  |
| --- | --- | --- | --- |
| CNT chirality | GR/PE | GR-SWCNT | GR-MWCNT |
| (0,0) | 51.03 | - | - |
| (6,6) | - | 74.97 | 68.01 |
| (8,8) | - | 79.85 | 75.35 |
| (10,10) | - | 77.93 | 72.10 |
